# Trust in Scholarly Communications and Infrastructure: Indigenous Data Sovereignty

**DOI:** 10.3389/frma.2021.752336

**Published:** 2022-01-12

**Authors:** Katharina Ruckstuhl

**Affiliations:** Otago Business School, University of Otago, Dunedin, New Zealand

**Keywords:** Indigenous data sovereignty, research infrastructure, decolonization, data governance, traditional knowledge, Nagoya Protocol, TK labels, metadata

## Abstract

Many Indigenous people have a deep mistrust of research, with some describing research as one of the “dirtiest” words in Indigenous language. The histories and experiences behind such mistrust are long and painful. Given what has been perceived as Indigenous objectification at the hands of largely Anglo-European others for research from which they fail to benefit, many communities now refuse research unless it is undertaken under certain, Indigenous-defined circumstances. Such refusal is a move away from others purposes and a move towards autonomy and self-determination. For some, this is a statement of sovereignty and it applies to all areas of endeavour, including the new frontiers of research and the structures that support them, such as datification of knowledge. This article examines data sovereignty from the perspective of Indigenous peoples. While data sovereignty has become a ubiquitous concern, Indigenous data sovereignty arises from contexts specific to Indigenous peoples. The focus of this article is to provide a brief overview of recent data sovereignty developments, along with the context that lies behind these activities. Through this examination, implications for trust in scholarly communications will be discussed.

## Introduction

Many Indigenous people have a deep mistrust of research, with some describing research as one of the “dirtiest” words in Indigenous language. The histories and experiences behind such mistrust are long and painful. Given what has been perceived as Indigenous objectification at the hands of largely Anglo-European others for research from which they fail to benefit, many communities now refuse research unless it is undertaken under certain, Indigenous-defined circumstances. Such refusal is a move away from others' purposes and a move toward autonomy and self-determination. For some, this is a statement of sovereignty and it applies to all areas of endeavor, including the new frontiers of research and the structures that support them, such as datification of knowledge.

This article examines data sovereignty from the perspective of Indigenous peoples, focusing on data held in government or state-funded research organizations. While data sovereignty has become a ubiquitous concern, Indigenous data sovereignty arises from contexts specific to Indigenous peoples. The focus of this article is to provide a brief overview of recent data sovereignty developments, along with the context that lies behind these activities. Through this examination, implications for trust in scholarly communication and infrastructure will be discussed.

The article proceeds as follows. The first section examines the impact of colonialism in relation to research derived from Indigenous people, their lands and genetic and cultural resources. Particular attention is paid to Indigenous notions of sovereignty, in contrast to nation-state or individual notions, from which is derived more recent call for Indigenous data sovereignty (IDS). I then look at the various contexts and infrastructures of data—administrative data held in government databases, biologically based data in biobanks held in research organizations, and data in collecting organizations such as galleries, libraries, archives and museums. This section identifies how Indigenous people are developing policies and processes for data sovereignty. Drawing on the previous sections, the final section considers implications for trust in scholarly communication and infrastructure, and the actions needed to engender trust.

## Data Sovereignty and Its Indigenous Context

### Colonialism and Sovereignty

With the increased digitization of all forms of information, how data is stored, attributed, categorized, organized, owned, managed and used has become a ubiquitous concern from the micro level of the individual to the macro levels of nations and global organizations. This avalanche of data and the ease in which it crosses borders has seen some call for data sovereignty. This ranges from calls for personal data sovereignty (Micheli et al., 2020) to proposed policies for the sovereignty of European data and digital infrastructure (EIT Digital, 2020).

There is no one definition of data sovereignty, although there are overlapping features, many of which relate to the rights of individuals, collectives or nations to have control and power over data whether within territorial locations or cross-jurisdictionally. Data sovereignty is also associated with privacy and the constraint of information flows, ownership, inclusiveness and the representation of different groups into decisions about how data is used or re-used (Hummel et al., 2021).

That sovereignty is the word to describe a desired solution to the problems associated with data resonates with Indigenous people, however not necessarily for the reasons found in others' use of the word. In their analysis of the discourse on digital sovereignty, Couture and Toupin (2019) note territorial authority and thus sovereignty had been an aim of the nation-state. However, the sovereignty ambition of nation-states has always been contested by other formations such as those of kinship, religion, tribe or feudal ties. Moreover, an absolutist position on sovereignty is increasingly bounded or limited by mechanisms such as international treaties, pacts and agreements, the activities of trans-global organizations, and global infrastructures such as telecommunications networks. Thus, while nation states may “imagine” they are sovereign (Anderson, 1983), this has often been more limited in practice.

It is in relation to nation states, and their imagined sovereignty over bodies, resources and territories, that Indigenous demands for data sovereignty arise. Before exploring this, it is important to note that it is always difficult and sometime perilous to define a group, including Indigenous peoples who are not homogenous. Such definitions can be fraught, particularly when constructed outside of Indigenous peoples' views of themselves (Corntassel, 2003). The United Nations (UN) has shied away from defining the word “Indigenous” preferring to “identify rather than define” Indigenous peoples (United Nations, 2006), with the following as common characteristics (Daes, 2008):

(a) Priority in time, with respect to the occupation and use of a specific territory;
(b) The voluntary perpetuation of cultural distinctiveness, which may include the aspects of language, social organization, religion and spiritual values, modes of production, laws and institutions;
(c) Self-identification, as well as recognition by other groups, or by State authorities, as a distinct collectivity; and
(d) An experience of subjugation, marginalization, dispossession, exclusion or discrimination, whether or not these conditions persist.

All of the above characteristics are worth considering in relation to IDS and the consequent flow-on effects into trust in scholarly communication infrastructure.

First, as (a) states, Indigenous peoples occupied and continue to inhabit specific territories. Many of these Indigenous people were subsequently displaced from or dispossessed of these territories, for the most part forcibly, despite in some cases such as in Aotearoa New Zealand, Canada and the United States, treaties being signed to maintain or share territories. Whether displaced from territory or not, the overwhelming experience for many Indigenous people is of marginalization and discrimination. This is not a matter of colonial pasts from which Indigenous people have moved on as they become subsumed or assimilated into nation states. It is a structured and ongoing reality for Indigenous people that manifests itself in socio-economic disparities and, for some, ongoing violence for territories over which Indigenous peoples consider they have rights and obligations.

Despite the past and ongoing efforts of some nation states to eradicate the cultures, languages and practices of Indigenous peoples, whether in the cause of the one sovereign nation, or whether to civilize and promote “development” of Indigenous people, distinct Indigenous cultures remain. Again, while it is fraught to essentialize cultures, as (c) in the UN identification suggests, there remain patterns of worldview and practice to which many Indigenous peoples ascribe. These include:

- Distinct knowledge systems, variously described for example as Indigenous knowledge (IK), traditional ecological knowledge (TEK) or folk knowledge. Such knowledges rather than primitive or pre-modern are characterized by dynamism and adaptation (Pool, 2015). Such knowledges also do not discount that which is spiritual or “revealed” knowledge, but rather use such knowledge alongside traditional and empirical knowledge (Dei, 2000);
- A distinct relationship to place to which Indigenous people have a sense of guardianship and protection for future generations, whether such generations are human or not (Colburn, 2021). From this relationship arises a sense not only of belonging but also connectedness, rights and obligations (Katerere et al., 2019);
- A collectivist rather than individualist approach to all facets of material life which can include how resources are used or distributed, who has the rights and obligations toward such resources, and how these resources are viewed such as being seen as “gifts” or “treasures” from creators (Colburn, 2021).
- Distinct languages through which knowledge, culture and relationship to place are transmitted intergenerationally despite 50% of the world's 6,500 languages under threat (Mackenzie and Davis, 2018)

In summary, Indigenous people have maintained their specific identities in a manner that that can be described as “survivance,” which is “more than survival, more than endurance or mere response …[but is] an active repudiation of dominance, tragedy, and victimry” (Vizenor, 1998, p. 13). This active repudiation extends to how Indigenous people have been positioned within nation-states through the plethora of laws, institutions, structures and infrastructures that maintain colonialism or settler colonialism (Gover, 2015). This includes the innumerable scholarly mechanisms associated with disseminating knowledge and research about Indigenous people, their lands, resources and cultures.

### Colonialism and Research

For many Indigenous people ‘research' has been, and for some, continues to be one of the “dirtiest” words in the Indigenous world's language (Smith, 2009). Hence there has been little trust in the research mission and the pursuit of generic knowledge and universal “truths” that are divorced from Indigenous lives, with research viewed as complicit in past and ongoing colonialism.

One emblematic example is James Cook's Transit of Venus voyage to the South Pacific in 1769, for which the Royal Society successfully raised £4000. Such funding was forthcoming not only out of scientific curiosity but through the Admiralty's secret instructions to Cook to discover unknown countries and gain knowledge of these to advance British trade. Meanwhile, the Royal Society proposed that gentleman botanist Joseph Banks convince the discovered savage and brutal nations of European superiority (Igler, 2019). While such attitudes were typical of the era, Banks' reputation rests on the collection of 30,000 botanical and over 1,000 animal specimens gathered during the three-year voyage. These were the first specimens from the South Pacific seen in Britain and catapulted Banks' career and prestige, leading to his eventual Presidency of the Royal Society and Directorship of the Royal Gardens at Kew (Agnarsdóttir, 2019).

Banks' vision for Kew was that it might become a botanical exchange house, whereby collectors would taxonomically name and then bring back new plants that were economically useful to expand the British Empire (Hopper, 2013). And indeed this is what occurred, with for example, Brazilian rubber and Andean cinchona bark from which quinine is made eventually transferred via Kew to start industries in Malaya and India respectively. Expropriation of such specimens and the Indigenous knowledge that went along with them, converted science knowledge into imperial economic power. The ongoing consequence of this is that nations from which Indigenous knowledge was acquired to identify the utility of a specimen, now pay ‘rents' in the forms of patents licenses or fees to access the biomedical or other technologies derived from these appropriated specimens (Brockway, 2011).

While Kew Gardens has recently acknowledged its role in Empire (Parveen, 2021), biocultural appropriation, as Brockway suggests, continues. This despite the Nagoya Protocol that, under the Convention of Biological Diversity, aims to provide a transparent legal framework for the fair and equitable sharing of benefits arising out of the use of a nation's genetic resources, including the traditional knowledge associated with genetic resources (United Nations, 2015). That such a protocol has been necessary speaks to the practices of what some call “biocolonialism,” which can be seen as a process whereby genetic resources from traditional medicines and seeds are altered sufficiently to render them patentable, thereby allowing corporations or research organizations to commodify and profit from the sale of such knowledge (Harry, 2011). As Tauli-Corpus has argued, Indigenous people do not understand the logic whereby plants and seed varieties developed and preserved over thousands of years by Indigenous people become “improved” in laboratories that then confers an intellectual property right to the “inventor” (Tauli-Corpus, cited in Whitt, 1998, p. 39).

Intellectual property regimes fail to protect collective Indigenous knowledge, hence retrospective global attempts, such as the Nagoya Protocol, to address this through access and benefit-sharing. That this continues to be an issue can be seen in disputes brought by Indigenous people around patenting attempts of Ojibwe wild rice, Mexican maize and Hawaiian taro (McGonigle, 2016). Even when pharmaceutical companies attempt to recognize TEK, such as Shaman Pharmaceutical's trade agreements with Amazonian peoples in the 1990s (McGonigle, 2016) or the South Africa's Council for Scientific and Industrial Research benefit sharing agreement with San (Vermeylen, 2007), the ultimate benefits, economic or otherwise, to Indigenous communities remain uncertain or negligible.

This brings us to the issue of patenting of other life forms and, particularly in our current context of the COVID-19 pandemic, ongoing research into the human genome. From an Indigenous perspective, the “promise” of genomic research to alleviate health problems is undercut by the experience of unethical practice and misuse of data (Jacobs et al., 2010). For example, the Havasupai Tribe of northern Arizona filed and won a lawsuit in 2010 against the Arizona Board of Regents over the misuse of their genetic samples, collected for research on type 2 diabetes in 1989 but subsequently used for studies on schizophrenia, ethnic migration, and population inbreeding—areas disapproved of by the original donors (Garrison, 2013). While informed consent is a central tenet of ethical practice in the human sciences, Reardon and TallBear (2012) argue that at least in the US context, when it comes to Indigenous populations, there is an overwhelming belief of the right to pursue science to advance universal knowledge. In such cases, Indigenous peoples acting to protect their own interests might be seen as hampering the knowledge commons. Such experiences are unfortunately common globally (Kowal et al., 2012).

A more recent example of this right to pursue knowledge involves the Institute for Development Research (IRD) in France, accused of biopiracy for patenting an anti-malaria drug without acknowledging the French Guianan indigenous community's traditional medicinal knowledge. As in the Havasupai case, the researchers initially saw themselves practicing a science based on the greater good, having collected the samples in 2009 “in good faith.” In this case, rather than a direct payment, the IRD agreed to a benefit-sharing arrangement with Guianan authorities as recommended under the Nagoya Protocol (Pain, 2016). While the European Union, of which France is a member, only legally adopted the Nagoya Protocol in 2014, the IRD's retrospective agreement indicates the increasing pressure from Indigenous groups for fair and equitable benefit. Without due diligence of the sources of genetic materials, European researchers can face fines of up to €810,000 and imprisonment. Currently EU interpretation of the Protocol excludes information stored in databases, however, this is under contestation and may change (V.O. Patents Trademarks, 2019).

Raw genomic data has emerged as a global commodity in the last few years, with research organizations increasingly interested in small populations, such as Indigenous people (Fox, 2020). Such commodification, and the historic harms to Indigenous people of which the Havasupai is but one example, have hastened Indigenous efforts to control how such data is accessed, stored and used. There have been calls not only for Indigenous-framed ethical approaches to consent, but also for greater oversight and governance of both the original genetic material and the data that is derived from the material. This then brings us to IDS which has gained popularity as a terminology more recently, but has been in the policies of some tribal groups such as Cherokee since at least the 1990s (Bardill, 2017).

Given the historic experience of many Indigenous people, data sovereignty is a form of “corrective justice” after several centuries of policies that marginalized and diminished the rights of Indigenous peoples (Tsosie, 2021). What distinguishes IDS is an emphasis on tribal or tribal nation self-determination and autonomous decision-making (Hudson et al., 2017), and a rebalancing of power relationships. Thus, while data sovereignty shares some of the concerns of the nation state to control flows of data, IDS is in fact a challenge *against* the nation state and its ontological foundations and presumptions (Moreton-Robinson, 2020). And while individuals may call for personal data sovereignty, particularly in relation to privacy, IDS pushes against a solely individualist approach to espouse *collective* principles based on long-held worldviews and practices (GIDA, 2019).

Simply put, IDS is the right of Indigenous peoples to control the collection, governance, ownership, and application of data about their people, lifeways, land and resources (Kukutai and Taylor, 2016). Where those data reside, as suggested above, is overwhelmingly in various non-Indigenous repositories, both public and private. How then, can data sovereignty be exercised, and what implications does this have for trust in scholarly communications and infrastructure?

## Recent Developments in Indigenous Data Sovereignty Policy and Practice

### Administrative Data

In the public sphere, statistical administrative data collected for government policy purposes often categorizes Indigenous people from the “5D” perspective, i.e., difference, disparity, disadvantage, dysfunction and deprivation. It is not the data itself that is the problem but the purposes for which such data are analyzed and then used. These data are often gathered from a research perspective that aggregates different tribal collectives, decontextualizes them from their social and cultural context and analyses Indigenous people as problematic in contrast to other groups (Walter and Suina, 2019). This “deficit” data analysis fails to take account of Indigenous priorities, values, culture, lifeworlds and diversity (Walter and Suina, 2019) or address Indigenous ability to develop their own nation-building aspirations (Rainie et al., 2017). Hence, an increasing Indigenous focus is on the collection and analysis of data that prioritizes Indigenous-defined objectives thereby reframing narratives of Indigenous people as deficient and lacking in some decontextualized comparative metric such as health, education, housing (Rainie et al., 2019). This more strengths-based or capability approach (Sen, 2001) posits Indigenous people as more than proficient at solving their own issues, provided State infrastructure and resources are equitably provided.

From a practice perspective, there are examples of administrative data being either co-constructed with or controlled by Indigenous people. For example, the Canadian OCAP^®^ principles of ownership, control, access, possession were a Canadian response to providing a framework for governance and statistical practices of health data. OCAP^®^ asserts Indigenous rights to control and benefit from their data with impacts on other national bodies and educational institutions that have likewise altered their data practices to empower Indigenous data control (Walker et al., 2017). Flow-on effects have included broader Indigenous-led research protocols, jurisdictional control and development of best practices for research using First Nations, Inuit and Métis data (Rowe et al., 2021).

### Biodata

As discussed previously in relation to genetic material there is a new Indigenous focus on not only ethical collection and consent but also secondary use of data (Garrison et al., 2020). Biobanks hold human biological materials and/or genetic information along with associated demographic and health information (Beaton et al., 2016). Given the global exchange of data, and the need to represent accurately population genetics to provide tailored health solutions, there is the need to include minority populations. One argument is that, contra to a belief that individuals are “gifting” their genetic biomarkers to help develop health breakthroughs such as precision medicine tools—a type of “public good”—there needs to be more of a focus on genetic stewardship. Such thinking arises from the observation that many Indigenous people fail to be the recipients of the proposed benefits of health innovations, even when the data is in the public domain. Hence there are increasing calls for either the development of Indigenous-controlled biobanks or for increased governance over existing biobanks (Tsosie et al., 2021).

There are a number of examples of good practice, where Indigenous groups and genetic researchers have developed positive working relationships, grounded in Indigenous worldviews of health (McWhirter et al., 2012) and targeted at developing Indigenous capacity and governance (McWhirter et al., 2015). Likewise, there are emerging examples of biobank data governance, for example, Aotearoa New Zealand's He Tangata Kei Tua, a culturally informed policy and practice for biobanks in relation to governance, operational, and community engagement activities (Beaton et al., 2016). Similarly, the four-year funded Canadian “Silent Genomes” project that, along with aiming to reduce health-care disparities and improve diagnostic success for children with genetic diseases from Indigenous populations, also aims to develop a First Nations governed background variant library as a reference to allow effective precision diagnosis (Garrison et al., 2019). Another example of Indigenous biobank control is the Native BioData Consortium created to keep Indigenous research samples and data within the provenance and governance of Indigenous communities (Tsosie et al., 2021).

Turning to plant materials, at the aforementioned Kew Gardens, there is now a recognition that imperialist views still prevail in relation to its collections, with scientists continuing to report how new species are discovered every year, despite the knowledge of and use of such plants for thousands of years by local people (Antonelli, 2020). It does not take much to find related views in academic publications. A 2020 article in the journal Antibiotics describes how “many students wrote their masters and PhD theses on ethnomedicinal uses by the Karen people” [an Indigenous hill tribe on the border of Myanmar and Thailand] but “strangely” did not focus on how Karen people's botanical knowledge was used to treat ailments like fever. Therefore, the author complied “the most comprehensive list to date of botanical species that are treated as therapies against fever by the Karen people… cover[ing] 25 Karen villages in Thailand and compiled a list that includes 125 species,” helpfully listing a taxonomy of the “high value plant species” on the open access mdpi site (Phumthum and Sadgrove, 2020). While the author does not claim to have discovered these plants, there is a “terra nullius” implication that Karen plant knowledge is “free” because the Karen do not have territorial sovereignty to the land on which the plants are found (Rojas-Páez and O'Brien, 2021). This carries on a mode of colonial thinking into science that was once used to dispossess many Indigenous people of their lands because it was “terra nullius” or “belonged to no one” (Harry, 2001).

However, there are also examples of scientists acknowledging that they are not the “discoverers” of new plants, with one Polish PhD student, Mateusz Wrazidlo, working with the Indigenous community of the Guiana Highlands to give a Pemón Arekuna name to an orchid species new to science. Wrazidlo states that this was aimed at “de-colonizing science nomenclature and giving more representation to indigenous [and] local languages” (Kimbrough, 2021). Such a practice embodies recent calls from ethnobiologists to decolonize institutions, projects and scholarship. The authors acknowledge that centralization of biocultural resources in Euro-American repositories and archives has been extractive and alienated Indigenous people from their cultural and biological heritage. Hence the authors recommend a set of practices that include repatriating biocultural collections to Indigenous stewards, ensuring that data around biocultural classifications accurately represents Indigenous understanding, showing reciprocal relationships in research rather than doing “parachute science” where researchers visit, collect and return to their home institutions, and respecting data sovereignty (Mcalvay et al., 2021).

### Data in Galleries, Archives, Libraries and Museums

Much tangible and intangible knowledge, in the forms of stories, songs and oral traditions resides in art galleries, libraries, archives and museums. While Indigenous people have been demanding repatriation of human ancestors and their cultural artifacts over many years, the reality is that institutions continue to hold vast Indigenous collections. There is an accelerating movement to incorporate Indigenous framed archival practices (Callison et al., 2016) and an acknowledgment of the role of such institutions in perpetuating colonialism (Giblin et al., 2019). At a structural level, there are well-documented cases of histories of racist and offensive subject terms and classification schemes that homogenize and essentialize and that have remained static, retaining their colonialist roots. Far from being neutral classifications, library taxonomies are inherently biased, reflecting the dominant perspective of the “other” (Vaughan, 2018) For example, Indigenous people do not classify themselves as “indigenous,” “native,” “aboriginal,” “Amer-Indian” or other such blanket description. As a Māori woman from Aotearoa New Zealand, I identify my tribal affiliations as Ngāi Tahu and Rangitāne. However, similar to government administrative data, library cataloging collectivizes groups of people to enable search, misnaming or using non-Indigenous terms to explain phenomena and maintaining a “rules-based” orientation to cataloging such as the Library of Congress, Dewey or Anglo-American Cataloguing Rules (Duarte and Belarde-Lewis, 2015). Such rules can be difficult to change, even when societal attitudes have.

One response to this has been to examine the metadata in archival classification systems. Metadata is the “data about data,” or the cataloging information about a collection. It describes information, it enables administrative functions to ensure data is stored, preserved and able to be accessed technically, it identifies rights e.g., copyright, and it structures disparate individual components into larger more meaningful understandings. As such it is ideologically based, and neither neutral nor objective but rather subjective in what it includes, omits or describes (Gartner, 2016; Haberstock, 2020). While user or “social”-generated, as opposed to archival specialist generated metadata is becoming more a feature (Alemu, 2018), Indigenous-generated metadata functions additionally to address colonial power structures.

In order to decolonize archival metadata, some institutions are participating with Indigenous groups to develop more nuanced metadata labels or “tags.” For example, in Aotearoa New Zealand librarians are adding Māori terms into subject headings, including authority files with Māori terms; instructions for faceting Western concepts such as “myths and legends” with Māori concepts of “history and genealogy”; and rules for faceting records to include the perspectives of the relevant tribes in a document (Duarte and Belarde-Lewis, 2015). In another project, Zuni elders worked with the A:shiwi A:wan Museum and Heritage Center to catalog Zuni items excavated in the 1920s. In this project, additional metadata schema were required to the “normal” to incorporate uses and practices of, and stories and narratives around objects (Haberstock, 2020). For some institutions, specificity about Indigenous material in collections can reveal a lack of knowledge, with metadata schema failing to associate content and the authorities of tribal nations, clans or families, their communities, or territories.

In a move similar to the repatriation of human remains or artifacts, Anderson and Christen (2019) advocate for “digital repatriation,” which cedes decision making about access, narration, curation, and circulation of research materials to the original stewards that in turn affects future documentation, recording, metadata, as well as publication. For them, attribution is key given that photographs, sound recordings, films, artworks and manuscripts documenting Indigenous lives are the property of the “author” under copyright law. This is similar to the way that an inventor who develops a treatment based on Indigenous medicinal knowledge can be granted a property right in the form of a patent. Given that authorship circulates in perpetuity through the infrastructures of research—catalogs, records, publications and citations—digital repatriation acts as a rupture to colonialism through re-attribution to and control by originating communities. The example that Anderson and Christen highlight is that of sound recordings of Passamaquoddy singers, recorded in the 1890s by ethnographer Fewkes without attributions but through interactions with descendants of the original singers, re-attributed to the individuals who supplied the voices. More than that, however, the Library of Congress record contextualizes the recording, includes cultural and traditional narratives supplied by the elder descendants and applies “Traditional Knowledge Labels” to the record, including one that indicates that the material is non-commercial.

Traditional knowledge labels (TK labels) are an emergent digital rights tool aimed at enabling Indigenous control over their materials in a context of increasing digitization of cultural heritage, its global circulation via the internet with varying degrees of open access, and third-party use of such material (Reijerkerk, 2020). Anderson and Christen have adapted the Creative Commons licensing approach that ameliorates against copyright to develop the Local Contexts platform (https://localcontexts.org/) that hosts TK licenses, labels and notices. The labels are designed to highlight that local Indigenous values and appropriate use remain embedded within archival materials, even if they have been outside community ownership for generations (Anderson and Christen, 2013). The labels themselves have been extensively trialed with Indigenous communities and can be applied to tribal archives to explicate access permissions internal to the tribe or externally to others who may find tribal cultural material online. To the TK labels have been added Biocultural (BC) Labels and Notices that operate in a similar way but for data derived from genetic resources to enhance the capacity for Indigenous control of Indigenous data (Anderson and Hudson, 2020). Additionally, they provide a visible machine-readable, persistent and durable connection between Indigenous communities and researchers, genetic resources, generated digital sequence information, and knowledge that exists as metadata in sample/data repositories and can appear on published articles (Liggins et al., 2021).

## Indigenous Data Sovereignty and Implications for Trust in Scholarly Communication and Infrastructure

TK and BC Labels are at the forefront of data stewardship and data governance models (van Geuns and Brandusescu, 2020) that globally have become urgent areas of enquiry, as explained at the start of this article. Indigenous enquiry additionally extends into areas such as:

- artificial intelligence and its potential to re-inscribe coloniality based on its original faulty data sets (Lewis, 2020);
- the critical examination of open access data standards such as the FAIR principles for scientific data management and stewardship, developed to enhance the ability of machines to automatically find and use research data and to supporting its reuse by individuals (Wilkinson et al., 2016). While the FAIR principles (Findable, Accessible, Interoperable, Reusable) allow for open access, such principles can be at odds with Indigenous positions in relation to certain types of tribal data. Hence, alongside FAIR, the CARE principles (Collective Benefit, Authority to Control, Responsibility, and Ethics) have been proposed. The principles describe high-level actions applicable within various data settings with a goal to implement CARE and FAIR across the data lifecycle in tandem (Rainie et al., 2020);
- Indigenous data provenance and the rules by which Indigenous peoples' data should be described and recorded. This current working group of the IEEE will make recommendations for metadata fields that can be used across industry sectors, including machine learning, artificial intelligence, contexts, biodiversity and genomic science innovation and other associated databases. This will include connecting data to people and place, and when appropriate, supporting future benefit sharing options (IEEE Standards Association, 2020).

IDS has ongoing implications for trust in scholarly communication and infrastructure. Indigenous people expect that at every level of the research lifecycle—from the accessing of raw data, whether qualitative or quantitative, to its storage in databases, biobanks or herbaria, and then onto its analysis, eventual publication and potentially secondary re-use of originating data—there will be policies and institutional practices that reflect the realities of Indigenous peoples, be useful for Indigenous purposes, and remain under Indigenous control, while promoting knowledge discovery and innovation (Rainie et al., 2020, p. 8).

For scholarly publishers, this is more than adopting diversity and inclusivity policies, although these are undoubtedly necessary (Dawson et al., 2020). It is also more than increasing Indigenous and other under-represented groups' accessibility to prestige publications, although this too is needed (Collyer, 2018). Rather, it is an examination of the “core” machinery of scholarly infrastructure—universities, ethics committees, funders, and others—of which data is increasingly its key component. Part of this examination and a consequent response may include applying digital rights management protocols, such as the TK and BC Labels and Notices, into publishing and related data management systems. For example, in 2020 ORCID, a not-for-profit software platform that provides a unique, persistent digital identifier to individual researchers ran a series of global webinars alongside the Global Indigenous Data Alliance and the US Indigenous Data Sovereignty Network to raise awareness of IDS. The webinars were an introduction to IDS aimed at research funders, institutions, publishers, and individual researchers (Akee et al., 2020). Following on from these webinars, ORCID is working with Local Contexts to create a “workflow” between the two organizations that enables researchers to request research or use existing Indigenous data. When the researcher is approved by the tribal group, the researcher's ORCID record will be updated with the metadata describing the work and tribal approval. This will enable Local Contexts to update a researcher's record to indicate they have permission or consent from the tribal group to conduct research or use the data.

Partnering with Indigenous groups, supporting conscientization of Indigenous issues, diversifying the workface are important, but they are insufficient. Research infrastructures need to move beyond the metaphoric rhetoric of “decolonization” (Tuck and Yang, 2012), to the actuality: making room for Indigenous decision-making and authority over their materials, wherever they may be located. Seen in this light, ORCID's approach to Indigenous data management is a core infrastructure response to IDS. It is a small but significant way by which Indigenous groups may have some control over access to and use of their own cultural, bio-cultural or genomic data. Potentially this then acts as a mechanism whereby provenance of such data is “on-the-record” and hence helps to identify those tribal groups that may need to be in discussions should benefits be eventually derived.

Indigenous trust in scholarly communication and infrastructure will be derived from the sum of the sets of activities described in this article. These include: reciprocal relationships; using Indigenous nomenclature and language; access and benefit-sharing arrangements; avoidance of “terra nullius,” “common good” or “universalist” thinking and methodologies that are then re-embedded in publications and open access data; global, national and institutional governance protocols and standards around Indigenous data; re-inscribing attribution and provenance into metadata; and using digital tools that reinforce Indigenous rights and stewardship.

As has been explained, IDS is but the latest field in a long history of Indigenous action to assert sovereignty. What is different now is that there are theories, tools, approaches and protocols that can be applied across a range of research infrastructure and settings to acknowledge and respond to Indigenous demands for data sovereignty. Non-response or inadequate response may lead to financial and reputational penalties, as the Havasupai and IRD examples suggest. Conversely, genuine efforts to apply IDS tools and methods can enhance reputation and trust. Given the newness of many of these tools and approaches, this will not be an easy or “quick-fix” process.

As the global breadth and depth of activity explained in this article suggest, demands for IDS in state and government run research organizations are increasing. The demand on private sector organizations such as academic publishers and the dissemination infrastructures they rely on are less well-canvassed, although no less pressing. Tools that have been developed to address IDS in the state sector, such as the TK and BC labels, may have relevance, as may policy and ethics approaches. However, it is too early to say to what extent these may be applicable, and further research in this area is warranted. What is clear is that organizations both public and private are increasingly asked to respond to the questions that IDS raises.

## Author Contributions

The author confirms being the sole contributor of this work and has approved it for publication.

## Funding

National Science Challenge SfTI Spearhead Project. 2019-S1-CRS Building New Zealands' Innovation Capacity.

## Conflict of Interest

The author declares that the research was conducted in the absence of any commercial or financial relationships that could be construed as a potential conflict of interest.

## Publisher's Note

All claims expressed in this article are solely those of the authors and do not necessarily represent those of their affiliated organizations, or those of the publisher, the editors and the reviewers. Any product that may be evaluated in this article, or claim that may be made by its manufacturer, is not guaranteed or endorsed by the publisher.
